# Co-Ingestion of Black Carrot and Strawberry. Effects on Anthocyanin Stability, Bioaccessibility and Uptake

**DOI:** 10.3390/foods9111595

**Published:** 2020-11-03

**Authors:** Celia Carrillo, Senem Kamiloglu, Charlotte Grootaert, John Van Camp, Marc Hendrickx

**Affiliations:** 1 Laboratory of Food Technology, Department of Microbial and Molecular Systems (M2S), KU Leuven, Kasteelpark Arenberg 23, B-3001 Leuven, Belgium; marceg.hendrickx@kuleuven.be; 2 Área de Nutrición y Bromatología, Facultad de Ciencias, Universidad de Burgos, Plaza Misael Bañuelos s/n, 09001 Burgos, Spain; 3 Laboratory of Food Chemistry and Human Nutrition (nutriFOODchem), Faculty of Bioscience Engineering, Department of Food Technology, Safety and Health, Ghent University, 653 B-9000 Ghent, Belgium; charlotte.grootaert@ugent.be (C.G.); john.vancamp@ugent.be (J.V.C.); 4 Science and Technology Application and Research Center (BITUAM), Bursa Uludag University, Gorukle, 16059 Bursa, Turkey; skamiloglu@uludag.edu.tr

**Keywords:** co-ingestion, anthocyanins, stability, bioaccessibility, uptake

## Abstract

Although the fate of anthocyanins along digestion has been a matter of research over the last decade, their bioaccessibility so far has been mainly assessed for single administered fruits or vegetables, which is far from the real scenario where they are co-ingested in a meal. Accordingly, the aim of this study was to evaluate the effect of simultaneous intake of fruit and vegetable on in vitro stability, bioaccessibility and uptake of anthocyanins. Black carrot and strawberry were used as food sources of anthocyanins. Anthocyanin identification and quantification were performed using HPLC-Qtof/HPLC-UV. Single matrices and mixtures thereof, were submitted to a standardized in vitro digestion procedure. Anthocyanin uptake was evaluated through an intestinal Caco-2 cell model. Our results showed an increased intestinal stability for specific anthocyanins as a consequence of co-digestion. The presence of the strawberry food matrix positively affected the bioaccessibility of the carrot associated cyanidin-based anthocyanins, whereas no reciprocal effect was observed for pelargonidin-based derivatives in the presence of the black carrot food matrix. Anthocyanin transport was maintained after co-administration. Overall, co-ingestion of black carrot and strawberry did not negatively affect the stability, bioaccessibility or uptake of cyanidin-based anthocyanins, although the effect on pelargonidin-based anthocyanins depended on the type of pelargonidin derivative.

## 1. Introduction

**Figure 1:**
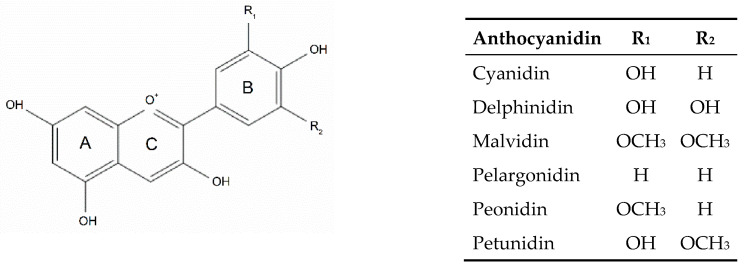
Anthocyanidin structures.

Anthocyanins are the compounds responsible for the purple, blue and red colors of various plant parts such as roots, flowers, fruits and leaves [1]. Chemically, anthocyanins are polyphenolic compounds that belong to the flavonoid family. They are structurally based on the polyhydroxy or polymethoxy derivatives of 2-phenylbenzopyrylium (flavylium ion). In plants, anthocyanins are found as glycosides of their respective aglycones, known as anthocyanidins. Anthocyanidins are composed of an aromatic ring A bound to a heterocyclic ring C that contains oxygen, also bound to an aromatic ring B through a carbon-carbon bond (Figure 1). Although more than 15 anthocyanidins have been identified, the most commonly found in nature are cyanidin, delphinidin, malvidin, peonidin, pelargonidin and petunidin. Cyanidin, delphinidin and pelargonidin, the nonmethylated anthocyanidins, constitute around 70% of colorful plant-based materials. A wide variety of anthocyanins (more than 700) have been reported in literature. They differ in the (i) number and position of the hydroxyl (OH) groups; (ii) methylation degree of the OH groups; (iii) nature, number and location of sugar moieties bound to the aglycone (arabinose, galactose, glucose, rhamnose, rutinose and xylose are frequently bound to anthocyanidins as mono-, di-, or trisaccharide forms; anthocyanins are mostly glycosylated in the 3-OH position and, to a lesser extent, in both the 3-OH and 5-OH positions); and (iv) presence of aliphatic or aromatic acids linked to the sugar moieties (sugar residues are commonly acylated with p-coumaric, caffeic, ferulic, sinapic, p-hydroxybenzoic, malonic, oxalic, malic, succinic or acetic acid) [2,3].

Next to their coloring attributes, anthocyanins show a broad diversity of biological activities as recently reviewed [4]. Briefly, anthocyanins are well-recognized free-radical scavengers and therefore their antioxidant capacity has been substantially explored. In addition, evidence has proposed that these bioactives potentially improve inflammatory processes in vitro and in vivo. Consequently, a role of dietary anthocyanins, and anthocyanin-based derivatives and metabolites, to combat obesity, to promote cardiovascular homeostasis and to improve cognitive function, together with several anticarcinogenic properties, have been reported based on in vitro and in vivo approaches [1,4].

However, when studying the role of bioactive compounds in human health, it is important to consider the amount that reaches their biological targets. In this sense, the term bioaccessibility refers to the fraction of a bioactive substance that is released from the food matrix after digestion and solubilized in the gut lumen for uptake across the intestinal mucosa, whereas bioavailability corresponds to the fraction of an ingested nutrient or compound that reaches the systemic circulation and the specific target, where it can exert its biological action [5,6]. Moreover, the non-bioaccessible or non-bioavailable compounds after digestion will follow the intestinal transit, being to some degree released and metabolized by the microbiota in the large intestine, where they can also generate important biological effects.

Lately, there has been a growing interest to better understand the digestive fate of food systems in order to strengthen the possible effects of nutrients on human health. Although clinical trials should be ideally applied to address the diet-related questions, there might be financial and ethical constraints, together with an interindividual variability in human responses, that even a “perfect” study design could not eliminate or completely overcome [7]. Therefore, in vitro digestion models simulating the gastrointestinal tract, have been proposed to study the bioaccessibility of bioactive compounds and nutrients. In spite of their simplicity, in vitro digestion models have been reported as useful tools in predicting the outcomes of the in vivo digestion [8]. Since a wide variety of in vitro digestion model conditions limit the ability to compare results among different studies, a standardized static in vitro digestion method has been recently proposed and applied to study the bioaccessibility of polyphenols from multiple dietary sources [9,10,11,12,13].

So far, the bioaccessibility of polyphenols, in most cases, has been assessed with single administered fruits or vegetables (or even from a purified compound) which is far from the real scenario where different fruits and/or vegetables are normally co-ingested in a meal. It is important to highlight that one of the factors that can affect the bioaccessibility of phytochemicals from plant-based sources is their interaction with other food components (together with the chemical/physical nature of the phytochemicals and food material microstructure), which influence the release of phytochemicals from food matrix [6]. In this respect, Gui-Fang et al. (2019) [14] found that the combination of fruits resulted in different interactions along the gastrointestinal tract that affected the total phenolic content recovered after digestion, which might imply that when different types of fruits are consumed simultaneously, the ultimate antioxidant capacity might not be in line with those to be expected from data on individual fruits. The co-digestion of red cabbage with different carotenoid-rich vegetables resulted in changes in the bioaccessibility of anthocyanins and carotenoids [15]. These recent works propose that there are interactions throughout constituents of different food sources during digestion, that lead to changes in the bioaccessibility of phytochemicals. Nevertheless, changes in the bioaccessibility of different individual anthocyanins as a result of co-ingestion of fruits and vegetables are still poorly explored, despite the importance of the issue bearing in mind the increasing offer of fruit and vegetables-based mixtures in the market.

The present study aims to report how the bioaccessibility of anthocyanins could be influenced by the co-ingestion of a fruit and vegetable mixture. A standardized in vitro digestion method, adequate for food polyphenols, was used. Selected anthocyanin-rich sources were black carrot and strawberry, respectively containing cyanidin-based and pelargonidin-based derivatives, since different stability properties have been attributed to these two families of compounds. Whereas the main pigment contained in strawberry is described to be remarkably prone to color loss, black carrot extracts provide high amounts of acylated structures that are known be stable at pH values that are unfavorable for some other anthocyanins [16,17]. This work aims to elucidate to what extent the co-ingestion of these two plant-based foods affects their own anthocyanin stability, bioaccessibility and uptake.

## 2. Materials and Methods

### 2.1. Plant Material

Fresh black carrots (*Daucus carota* L. ssp. *sativus* cv. *atrorubens* Alef) and strawberries (*Fragaria x ananassa*, cv. Elsanta) were purchased from Turkey and Belgium, respectively. The samples were grounded in liquid nitrogen, subsequently freeze dried and stored at −80 °C until use.

### 2.2. Experimental Set-Up

First, mass spectrometric identification of black carrot and strawberry anthocyanins was developed. Then, to evaluate the effect of the co-ingestion of black carrot and strawberry in terms of the fate of anthocyanins under gastrointestinal conditions, (i) the anthocyanin gastrointestinal stability, (ii) in vitro bioaccessibility and (iii) transepithelial transport were assessed in both the single samples and a mixture thereof (performed in order to reach a similar anthocyanin content from each matrix and therefore avoid concentration dependent effects). This involved (i) monitoring the gastrointestinal stability of these compounds in black carrot and strawberry extracts incubated with simulated gastric and pancreatic juices, (ii) determining their recovery following in vitro digestion of the powdered sample (i.e., compounds naturally involved in their inherent food matrix) and (iii) assessing their absorption through a Caco-2 cell model in both a single of mixed setup.

### 2.3. Anthocyanin Chemical Extraction from Undigested Samples

Anthocyanin extraction from lyophilized powdered samples was carried out according to a previously published method [18]. Briefly, 5 mL of 75% aqueous methanol with 0.1% formic acid was added to 0.1 g of sample. The samples were then sonicated for 15 min in an ultrasonic bath (Elma S60H elmasonic, Singen, Germany) for 15 min, followed by centrifugation for 10 min, at 3000× *g* and 4 °C (Sigma Laboratory Centrifuge 4K15, Osterode am Harz, Germany). The collected supernatants were transferred to a clean tube and the extraction procedure was repeated for the remaining pellet. The two consecutive supernatants were combined to a volume of 10 mL and kept at −20 °C until analysis. Black carrot and strawberry extracts used in cell culture assays were prepared as previously described [19]. Briefly, the prepared extracts were dried under nitrogen and prior to toxicity test and transport experiments, extracts were dissolved in Hank’s balanced salt solution (HBSS; Gibco, Life Technologies; Carlsbad, CA, USA).

### 2.4. Identification and Quantification of Anthocyanins

Anthocyanin analysis was performed as described elsewhere [13]. The anthocyanin content was quantified using a BioL-system (Dionex, Sunnyvale, CA, USA) with an AD25 UV-Vis detector (set at 520 nm). To separate the different anthocyanins, a polymer reversed phase column (PLRP-S; 250 × 4.6 mm, 5 µm particle size, 100 Å pore size; Varian, Palo Alto, CA, USA) was used. The separation was performed by applying a gradient elution (1 mL/min) at 25 °C with 4% phosphoric acid in water (eluent A) and 60% acetonitrile (eluent B). The conditions were set as: 0–10 min (10% B); 10–15 min (increased to 18% B); 15–46.5 min (increase to 25% B); 46.5–51.5 min (increase to 100% B); 51.5–56.5 min (100% B); 56.5–61.5 min (decrease to 10% B); 61.5–66.5 min (10% B). The quantification was based on calibration curves of cyanidin-3-*O*-glucoside or pelargonidin-3-*O*-glucoside, depending on the type of aglycone. The total anthocyanin content was calculated as the sum of the individual anthocyanin concentrations and expressed as mg/g dry weight (dw) of cyanidin-3-*O*-glucoside/pelargonidin-3-*O*-glucoside equivalents.

Anthocyanin identification was performed by liquid chromatography time-of-flight (TOF) mass spectrometry, following the procedure described elsewhere [13]. The mass spectrometer was equipped with a Jetstream electrospray ionization (ESI) source and positive electrospray ionization mode was used for data acquisition. The source parameters were: sheath gas temperature and flow: 350 °C and 11 L/min, respectively; nebulizer pressure: 45 psi; capillary voltage: 100 V; skimmer: 45 V; drying gas temperature and flow: 325 °C and 10 L/min, respectively. The mass spectrometer was set to acquire over *m*/*z* 20–1600. The identification of the compounds was performed by comparing their mass with data available in literature.

### 2.5. In Vitro Digestion Procedure

A standardized in vitro digestion procedure was applied to each sample [20]. For the gastric phase, 7.5 mL of a simulated gastric fluid (in mM: KCl 6.9, KH_2_PO_4_ 0.9, NaHCO_3_ 25, NaCl 47.2, MgCl_2_(H_2_O) 0.1, (NH_4_)_2_CO_3_ 0.5, pH 3.0), 5 µL of CaCl_2_ (0.3 M) and 1.6 mL of porcine pepsin solution (25,000 U/mL) were added and the pH of was adjusted to 3.0 using HCl (1 M). Demineralized water was added to the gastric chyme to complete a volume of 20 mL. Nitrogen was flushed into the headspace of the tubes before incubation at 37 °C during 2 h in the dark, while end-over-end rotation (40 rpm). For the intestinal phase simulation, the gastric chyme was mixed with 11 mL of simulated intestinal fluid (in mM: KCl 6.8, KH_2_PO_4_ 0.8, NaHCO_3_ 85, NaCl 38.4, MgCl_2_(H_2_O) 0.33, pH 7.0), 40 µL of CaCl_2_, 5 mL of pancreatin solution (800 U/mL based on trypsin activity) and 2.5 mL of bile solution (160 mM). NaOH (1 M) was used to adjust the pH to 7 and water to complete the volume until 40 mL. Prior to incubation in the dark, at 37 °C, 2 h, end-over-end rotation, the headspace of the tubes was flushed with nitrogen.

#### 2.5.1. Gastric and Intestinal Stability Calculation

Aliquots from gastric and intestinal digests were sampled along black carrot and strawberry extracts digestion, stabilized by acidification with formic acid and stored at −20 °C until further analysis. Stability (%) was calculated as indicated by Equations (1) and (2).

(1) $$Gastric\;stability\;\left(\%\right)=\frac{\left(anthocyanin\;\left(\mathrm{mg}\cdot 100{\;\mathrm{mL}}^{-1}\right)\;after\;gastric\;digestion\right)}{\left(anthocyanin\;\left(\mathrm{mg}\cdot 100{\;\mathrm{mL}}^{-1}\right)\;before\;digestion\right)}\times 100\;$$

(2) $$Intestinal\;stability\;\left(\%\right)=\frac{\left(anthocyanin\;\left(\mathrm{mg}\cdot 100{\;\mathrm{mL}}^{-1}\right)\;after\;intestinal\;digestion\right)}{\left(anthocyanin\;\left(\mathrm{mg}\cdot 100{\;\mathrm{mL}}^{-1}\right)\;before\;digestion\right)}\times 100$$

#### 2.5.2. Bioaccessibility Calculation

After digestion of the powdered samples, an ultracentrifugation (Beckman L7 ultracentrifuge, Palo, Alto, CA, USA) at 165,000× *g*, 4 °C, 65 min, was performed to separate soluble and non-soluble fractions. The supernatant, containing the bioaccessible anthocyanins, was isolated. For the determination of anthocyanins, the digest was acidified with formic acid (1%) to stabilize the compounds, prior to the quantification by high performance liquid chromatography (HPLC). Results are shown as the ratio of the bioaccessible compound concentration to the initial compound concentration in the sample, as indicated in Equation (3).

(3) $$Bioaccessibility\;\left(\%\right)=\frac{\left(anthocyanin\;\left(\mathrm{mg}.100{\;g}^{-1}\right)\;after\;digestion\right)}{\left(anthocyanin\;\left(\mathrm{mg}.100{\;g}^{-1}\right)\;before\;digestion\right)}\times 100$$

### 2.6. Maintenance of Cell Culture

The human colon adenocarcinoma Caco-2 cells (HTB37TM) from American-type culture collection (ATCC; Manassas, VA, USA) were maintained in 25 cm^2^ canted neck tissue culture flasks (Sarstedt Co., Essen, Belgium) using high-glucose Dulbecco’s Modified Eagle Medium (DMEM) supplemented with Glutamax (Gibco, Life Technologies, Carlsbad, CA, USA), 10% heat-inactivated fetal bovine serum (FBS; Greiner Bio One, Wemmel, Belgium), 1% penicillin streptomycin (Pen Strep; Gibco, Life Technologies), and 1% nonessential amino acids (NEAA, Gibco, Life Technologies). Cells were grown at 37 °C in a humidified atmosphere of 10% CO_2_ in air (Memmert CO_2_ incubator, Memmert GmbH & Co., Nurnberg, Germany) and at 80–90% confluence they were subcultured. For subculturing procedure, cells were washed with 3 mL phosphate buffered saline (PBS; Gibco, Life Technologies), detached from the flask with 2 mL of 0.25% trypsin-EDTA wash (Sigma-Aldrich, Steinheim, Germany) and incubated for 3–5 min at 37 °C and 10% CO_2_ in air. A certain volume of suspension was seeded into new flasks and the final volume was made up to 4 mL with the DMEM medium with Glutamax, 10% FBS, 1% Pen Strep, and 1% NEAA.

### 2.7. Toxicity Test

Caco-2 cells seeded in 96-well plates were differentiated for 14 days after confluency and treated with various concentrations of black carrot and strawberry extracts, as well as their mixture. After 4 h of incubation, the cytotoxic effects of black carrot and strawberry samples on Caco-2 cells were assessed using the 3-(4,5-dimethylthiazol-2-yl)-2,5 diphenyl tetrazolium bromide (MTT) test. In total, 50 µL MTT (Sigma-Aldrich) dissolved in PBS (5 mg/mL) was placed in 100 µL culture medium and incubated at 37 °C for 2 h to allow its conversion to formazan. After 2 h, the medium was discarded to dissolve formazan crystals in dimethyl sulfoxide (DMSO), and absorbance was measured with a Bio-Rad multiplate reader (Bio-Rad Laboratories, Hercules, CA, USA) at 570 nm.

### 2.8. Transport Experiments

For transport experiments, 6-well Transwell^®^ plates (0.4 μm pore diameter, 24 mm insert, Corning Costar Co., Elscolab, Kruibeke, Belgium) were seeded with Caco-2 cells at a concentration of approximately 6.0 × 10^5^ cells per well. Culture medium with and without cells were transferred to the apical chamber and basolateral chambers in volumes of 2 and 2.5 mL, respectively. After seeding, for 21 days, cells were allowed to grow and differentiate into confluent monolayers. Culture medium was refreshed thrice a week. Experiments were conducted using the maximum nontoxic concentrations of black carrot and strawberry extracts, and their mixture. Transepithelial electrical resistance (TEER) values were measured on the day of the transport experiment (day 21) before, during, and after (t = 0, 4, 24 h) the experiments with an automated tissue resistance measuring device (REMS, World Precision Instruments, Hertfordshire, UK). The transport medium was replaced with HBSS, and preincubated for 1 h. The pH of the samples in HBSS was adjusted to the pH of the duodenum, which is 6.5, filter sterilized and placed to the apical chamber of the culture wells (2 mL). Extract-free HBSS was adjusted to pH 7.5, sterilized and placed to the basolateral chamber (2.5 mL). Caco-2 cells were treated with samples at 37 °C and 10% CO_2_ for 4 h. Samples were taken from both apical and basolateral chambers every 2 h and stabilized by reducing the pH to 2 using formic acid, and kept at −20 °C before analysis.

### 2.9. Statistical Analysis

One-way analysis of variance (ANOVA), followed by a least-significant-difference (LSD) test (*p* < 0.05 being considered statistically significant) were performed to determine differences between mean values (*n* = 3), by using the software Statgraphics Centurion XVI.

## 3. Results and Discussion

### 3.1. Identification and Quantification of Black Carrot and Strawberry Anthocyanins

The total anthocyanin content of black carrot and strawberry was (mg/g dw) 17.01 ± 0.01 and 9.38 ± 0.02, respectively (Table 1). Total anthocyanin content can vary considerably depending on the variety, region of cultivation, maturity of the fruit and the solvent used to extract the anthocyanins. Although there are limitations in the studies that compare absolute anthocyanin concentrations, our results are within the range reported by other authors and confirm both black carrot and strawberry as anthocyanin rich sources [13,18,21].

**Figure 2:**
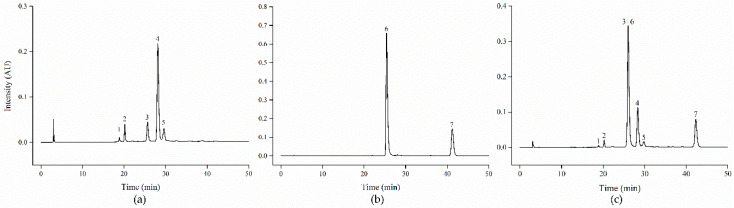
HPLC separation of anthocyanins (520 nm) from a black carrot extract (**a**) strawberry extract (**b**) and their mixture (1:1, *v*:*v*) (**c**). For peak assignment see Table 1.

Five major anthocyanins were identified in black carrot (Table 1; Figure 2a). All of them are cyanidin-based anthocyanins with different sugar moieties, where three are acylated with sinapic, ferulic and coumaric acids (peaks 3, 4, 5, respectively), whereas two of them are non-acylated anthocyanins (peaks 1 and 2). 89% of the total anthocyanins contained in black carrot presented an acylated structure, and the ferulic acid derivative of cyanidin-3-xylosyl-glucosyl-galactoside stood out as the major anthocyanin in this plant material (around 65% of total anthocyanins). The anthocyanin profile found in the present study is in agreement with those reported for black carrot elsewhere [13,19,22,23].

In the strawberry sample, two pelargonidin based anthocyanins were identified (Table 1; Figure 2b), which is in agreement with previous reports showing that anthocyanin-rich fruits can be divided into different groups based on the types of aglycones of their anthocyanins. Strawberry stands out as the main representative to contain pelargonidin-based anthocyanins [24]. Although differences in the anthocyanin profile among strawberry cultivars have been extensively reported [25], our results showed that pelargonidin-3-glucoside (peak 6) was the predominant anthocyanin, accounting for the 76% of total anthocyanins in this strawberry sample, followed by pelargonidin-3-malonyl-glucoside (peak 7), which is in line with previous studies [21,26,27]. Small amounts of cyanidin-3-glucoside have been also found in cultivar Elsanta, although this cyanidin has been reported to be influenced by temperature, growing conditions or latitude [28].

Figure 2c show the HPLC-DAD chromatogram of the black carrot and strawberry mixture. Cyanidin-3-xylosyl-sinapoyl-glucosyl-galactoside (peak 3) coeluted with pelargonidin-3-glucoside (peak 6). Different attempts for gradient optimization did not allow separating these two anthocyanins, which is in agreement with previous reports using even a different column and chromatographic conditions [29,30]. Since in this mixture the combined peak is mostly constituted of pelargonidin-3-glucoside, we will further list it as pelargonidin-3-glucoside. Results will be interpreted with caution accordingly.

### 3.2. Stability of Black Carrot and Strawberry Anthocyanins, Effect of Co-Digestion

The stability of black carrot and strawberry anthocyanins to gastric and intestinal conditions is presented in Table 2.

Gastric recoveries of 59% and higher were found for the anthocyanins assessed in the present study. Our results are in agreement with previous studies performed in different fruit and vegetable sources, such as red cabbage, purple sweet potato, pomegranate or red grapefruit (reported anthocyanin gastric stabilities: 72–110%) [31,32,33,34]. The recovery values in gastric fluid are anthocyanin specific. Significant differences (*p* < 0.05) between black carrot and strawberry overall anthocyanin stabilities were observed (82% and 90%, for black carrot and strawberry anthocyanins, respectively). The stability of black carrot anthocyanins to gastric conditions varied from 59% to 86%, depending on the type of cyanidin. The highest stability was observed for the ferulic acid derivative, and the lowest for the non-acylated derivative cyanidin-3-xylosyl-glucosyl-galactoside. Acylated anthocyanins have been reported to be more stable to temperature and pH changes than nonacylated ones because of intramolecular copigmentation effects of acylated anthocyanins [35], although the type of acylated groups may also affect the stability of anthocyanins in digestion [34].

Previous studies performed experiments in both the presence and absence of pepsin and observed similar results for all the phenolic compounds assessed, which indicates that the observed effects were mostly attributable to the chemical conditions of the test [36]. In aqueous solution, anthocyanins undergo structural re-arrangements as a consequence of pH changes, leading to equilibria between four molecular structures: quinoidal base (blue), flavylium cation (red), carbinol (colorless) and chalcone (yellowish) forms [3]. Thus, it has been reported that the stability of anthocyanins under gastric conditions is most likely due to acid conditions during the gastric phase under which anthocyanins are found in the flavylium cation stable form [2,37].

In turn, our results showed a decrease in the anthocyanin stability under intestinal conditions (mean recovery of total anthocyanins 86% and 53%, after gastric and intestinal conditions, respectively). The low recovery of anthocyanins after intestinal conditions could be partially due to the transformation of the flavylium cation to the colorless chalcone pseudobase at the pH of the intestinal environment. The colored flavylium cation form predominates at pH below 3, but at pH > 4–5 anhydrobases (quinoidal, carbinol and chalcone) become gradually more stable and increasingly formed as pH rise [38,39]. Therefore, anthocyanins may be converted into some noncolored forms, oxidized, or degraded into other compounds. Moreover, several authors claimed that the loss may be partly caused by the co-precipitation of anthocyanins with insoluble complexes with particulates or components of the pancreatin/bile salt mixture that arise during acidification of the digest [39].

Significant differences between black carrot and strawberry anthocyanin intestinal recoveries were observed, with a higher stability for cyanidin-based anthocyanins (59% and 48%, for black carrot and strawberry, respectively) under these conditions. Previous research has shown that the major strawberry pigment is extremely prone to color loss, whereas black carrot extracts provide an important amount of acylated structures that have been described to be stable at pH values otherwise unfavorable for anthocyanins [16,17]. However, it is important to highlight that non-acylated derivatives showed the highest stability to intestinal conditions, in both black carrot and strawberry extracts. Although it has been reported that in general, acylated anthocyanins are more stable than nonacylated forms, several authors proposed that the acylations may be cleaved along the digestive procedure, which may also be valid for our study. We found that the overall recovery of cyanidin-3-xylosyl-glucosyl-galactoside along the gastrointestinal tract was significantly higher than cyanidin-3-xylosyl-feruloyl-glucosyl-galactoside and cyanidin-3-xylosyl-coumaroyl-glucosyl-galactoside. Therefore, it is suggested that cyanidin-3-xylosyl-glucosyl-galactoside might be obtained by removal of the feruloyl and coumaroyl from cyanidin-3-xylosyl-feruloyl-glucosyl-galactoside and cyanidin-3-xylosyl-coumaroyl-glucosyl-galactoside, respectively. A similar approach behind pelargonidin-based anthocyanin could apply in the case of strawberry, with a cleavage of the malonyl group along digestion, as previously suggested by other authors [10]. Our findings confirm the results of an in vivo study carried out with healthy volunteers taking black carrot juice [40], were nonacylated anthocyanins were found to be more bioavailable than acylated anthocyanins.

There was no significant difference between cyanidin-based anthocyanin gastric recovery values in absence or presence of the strawberry extract. In contrast, the gastric stability of pelargonidin-based anthocyanins altered significantly when digested in combination with the black carrot extract, with a recovery decrease or increase for the non-acylated or acylated-pelargonidin, respectively. Co-digestion of black carrot and strawberry extracts resulted in a significant increase in the intestinal recovery of all the anthocyanins with exception of cyanidin-3-xylosyl-coumaroyl-glucosyl-galactoside, that maintained its inherent intestinal stability, and pelargonidin-3-glucoside that was negatively affected by co-digestion. These results imply that the effect of co-digestion seems to be anthocyanin specific. Several authors argued potential interaction between the anionic anthocyanins and cationic components. The chelation capacity of anthocyanins can form stable complexes with metal ions, resistant to the gastric/intestinal digestion, although these complexes were dependent on the anthocyanin structure and induced either increases or decreases in the recoveries [41]. Whether remnant cations present in the extracts could be behind the increases observed should be a matter of further research. In addition, the probability that some components may be protected in complex mixtures of polyphenols needs further consideration.

### 3.3. In Vitro Bioaccessibility of Black Carrot and Strawberry Anthocyanins and Effect of Co-Digestion

Although the gastrointestinal stability of black carrot and strawberry anthocyanins have been described in detail in the previous section, the bioaccessibility is crucial in terms of their health functionality. As discussed in the introduction, the food matrix plays a crucial role in terms of bioaccessibility.

In this sense, the recovery values representing the bioaccessibility obtained after in vitro digestion of the raw plant material (Table 3) are lower than those discussed for the extracts (Table 2). This confirms that the food matrix is a limiting factor in the bioavailability of food bioactives. However, the black carrot matrix seems to affect the bio-accessibility to a higher extent, since a mean decrease of 25% and 13% was observed for black carrot and strawberries anthocyanins, respectively (when the food matrix was included in the digestion procedure). Several authors have studied the role of structural barriers in anthocyanin bioaccessibility, and argued that cell wall disruption will enhance the release of the anthocyanins, but at the same time anthocyanins might interact with dietary fibers leading to two counteracting factors that modulate their bioaccessibility [13]. Dietary fibers constitute important carriers of phenolic compounds and for that reason, have an effect on their bioaccessibility, as fiber-entrapped polyphenols are poorly extracted and scarcely soluble in the gastrointestinal fluids [42,43]. Therefore, our results might be explained by the different composition of the two food matrices under study, with a higher dietary fiber content in case of black carrot. This is in agreement with several reports showing that foods with higher fiber content caused lower bioaccessibility of polyphenols [43].

In previous work [13] we showed that although overall anthocyanin bioaccessibility increased twice when the compounds were released from the matrix and passed into an aqueous medium (the bioaccessibility of anthocyanins would be maximized in the form of juices), the degradation of anthocyanins in the gastrointestinal tract might be the main factor limiting their bioaccessibility. Our findings are in line with this statement, since cyanidin-3-xylosyl-glucosyl-galactoside and cyanidin-3-xylosyl-coumaroyl-glucosyl-galactoside presented the highest and lowest bioaccessibility values, respectively, in clear connection with their stabilities under intestinal conditions. As discussed above, intestinal conditions may cause cleavage of cyanidin-3-xylosyl-feruloyl-glucosyl-galactoside and cyanidin-3-xylosyl-coumaroyl-glucosyl-galactoside to cyanidin-3-xylosyl-glucosyl-galactoside.

Therefore, our results confirm that bioaccessibility values observed for strawberry and black carrot anthocyanins represent a combined effect of their limited intestinal stability and their interaction with insoluble compounds originating from the food matrix. A wide range of bioaccessibility values for anthocyanins have been found in literature (17–59%) [10,13,44]. The chemical structure of the anthocyanins, their intestinal stability, their interaction with the food matrix or the in vitro digestion methodology applied could be the primary reasons for this variation.

Co-digestion of black carrot and strawberry resulted in a significant increase of black carrot anthocyanins bioaccessibility. However, different effects were observed for strawberry anthocyanins. While co-digestion did not affect pelargonidin-3-malonyl-glucoside bioaccessibility, a significant decrease in bioaccessibility was observed in the case of pelargonidin-3-glucoside. This might indicate that (i) the black carrot food matrix components resulted in a different effect depending on the type of anthocyanin, with cyanidins and the non-acylated pelargonidin being more susceptible to fiber-interaction, since pelargonidin-3-malonyl-glucoside was not affected in the presence of black carrot matrix and/or (ii) strawberry food matrix components may protect cyanidins under gastrointestinal conditions, increasing therefore their bioaccessibility. Several authors have also observed that the stability of anthocyanins during intestinal digestion varies strongly depending on food matrices and some components of red cabbage matrix have been also hypothesized to protect more labile anthocyanins [45], which reinforces our findings. In addition, a recent review which summarizes some general characteristics important in polyphenol-fiber interactions (a.o. polyphenol and fiber chemical structures or environmental conditions), conclude that when foods are taken in combinations in a meal, the complexity of these mixtures will influence the dietary fiber-polyphenol associations when compared to the same foods taken individually [43].

In general, acylated anthocyanins and 3,5-diglycosides are more stable than nonacylated forms and 3-monoglycosides, respectively; higher methoxylation of aglycone hydroxyls increases stability whereas anthocyanins with more hydroxylated aglycones present lower stability [39]. Nevertheless, these principles are not always observed in our study. In fact, pelargonidin derivatives were not always more stable than cyanidin (more hydroxylated) derivatives, and no consistent effect was observed for glycosyl groups, since the effects shown depend on the conditions applied—gastric versus intestinal—together with the presence or absence of food matrix along the digestion procedure. However, our results highlight that the non-acylated cyanidin with a higher number of glycosyl derivatives—cyanidin-3-xylosyl-glucosyl-galactoside—is the most bioaccessible anthocyanin. Thus, although cyanidin-3-xylosyl-glucosyl-galactoside did not show the highest stability under gastric conditions, it presented an overall higher recovery independently of the presence of its inherent food matrix or even other food matrices along the digestion.

### 3.4. Transport of Black Carrot and Strawberry Anthocyanins through Intestinal Caco-2 Cells. Effect of Co-Administration

**Figure 3:**
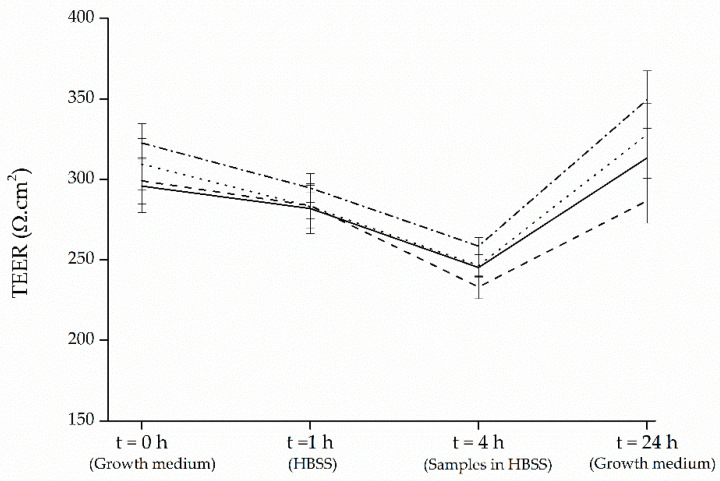
TEER (transepithelial electrical resistance) measurements carried out in growth medium (Dulbecco’s Modified Eagle Medium, DMEM, with Glutamax, 10% fetal bovine serum, FBS, 1% Pen Strep and 1% nonessential amino acids, NEAA) and during incubations in Hank’s Balanced Salt Solution (HBSS). Data represent mean values ± SD (3 biological and 3 technical replicates). Solid line corresponds to the blank (no treatment); dashed line to black carrot; dotted line to strawberry; dashed-dotted line to the mixture black carrot-strawberry.

Prior to performing transport experiments, MTT assay, evaluating the cellular viability related to mitochondrial activity, was performed to ensure that the extracts are intoxic to Caco-2 cells. Additionally, Caco-2 cell monolayer integrity was tested using TEER measurement and only monolayers with a TEER value above 300 Ω.cm^2^ were utilized for the transport experiments. The TEER measurements applied to examine the integrity of monolayers showed that initial TEER values were mostly retained (≥91%) as a result of pre-incubation of Caco-2 cells with HBSS for 1 h (Figure 3). However, treatment of cells with the black carrot and strawberry samples for 4 h resulted in reductions of TEER values (17−22%), which were restored after 24 h (≥96% of initial values), suggesting that the decrease in integrity of cell layer was not irreversible. Moreover, even though the TEER values reduced after transport, yet they were above the acceptable minimum level indicated in the previous studies (200 Ω.cm^2^) [46].

**Figure 4:**
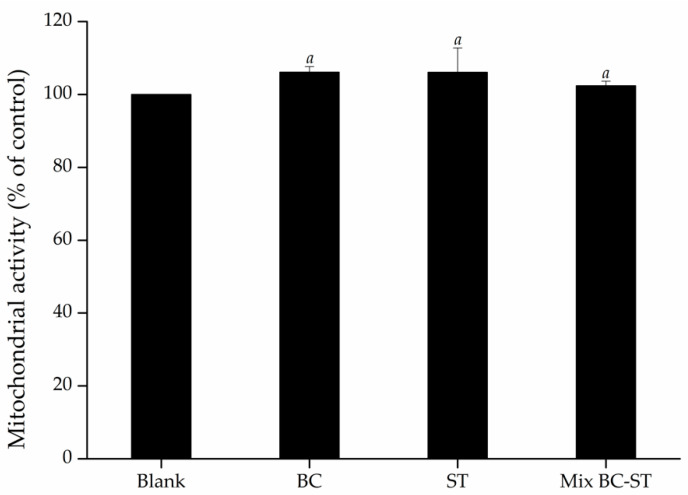
Mitochondrial activity (measured using MTT assay) of Caco-2 cells exposed to black carrot (BC), strawberry (ST) and their mixture (Mix BC-ST), expressed as % of control (no treatment). Different letters indicate significant differences.

Along with the measurement of TEER, to ensure that the selected concentrations of samples were intoxic to Caco-2 cells, MTT assay, which measures the cellular viability related to mitochondrial activity, was also performed after 24 h of treatment (Figure 4). The results of MTT assay demonstrated that none of the samples reduced cell viability compared to cells that are not treated.

The anthocyanin contents of the non-toxic concentrations of black carrot and strawberry samples applied on intestinal Caco-2 cells are given in Table 4 (time = 0 h). The anthocyanin recovery on the basolateral chamber at two different time points (t = 2 and 4 h) were presented as the percentage of the initial amount of anthocyanin placed to the apical chamber at t = 0 h. As shown in Table 4, anthocyanins could be transported through Caco-2 monolayers in intact glycone form. Anthocyanin uptake following 2 h of exposure to samples was relatively low (≤2.5%), and some compounds including cyanidin-3-xylosyl-glucosyl-galactoside and pelargonidin-3-malonyl-glucoside were even not detected. These findings are comparable with previous studies [19,27]. After 4 h, anthocyanin transport increased significantly (1.9–9.1%). Moreover, for all samples, the transport of nonacylated anthocyanins was greater than the acylated anthocyanins, which is in agreement with an in vivo study carried out with participants consuming black carrots [47] and which showed that the urine and plasma nonacylated anthocyanin concentrations were notably higher compared to acylated anthocyanins. Addition of the black carrot and strawberry mixture did not significantly affect cyanidin and pelargonidin uptake (*p* < 0.05). On the other hand, co-administration of anthocyanin-rich blueberry and grape extracts revealed 3 to 5-fold increased accumulation of blueberry phenolic metabolites in plasma and decreased their presence in feces to the same extent, although no reciprocal interactions were observed for grape phenolics [48].

Despite the fact that the anthocyanin absorption mechanism in the intestine is uncertain, it has been suggested that anthocyanins could modulate the transporters responsible for their transport. Glucose transporters are the candidates for anthocyanin transport, as they have a sugar moiety, particularly a glucose residue. The major hexose transporters reported in Caco-2 cells are the sodium dependent glucose transporter 1 (SGLT1) and glucose transporter 2 (GLUT2) [49]. Accordingly, a study [50] indicated that treatment with anthocyanin rich berry extract derived from berries including strawberry reduce SGLT1 and GLUT2 expressions. Some studies proposed that the quick appearance of anthocyanins in plasma after ingestion could be due to their transport through the gastric wall. Cell culture studies demonstrated that anthocyanins could cross the monolayers of MKN-28-differentiated adenocarcinoma stomach cells [51]. Consequently, the poor bioavailability of anthocyanins may be attributable to their substantial presystemic metabolism, instead of low absorption from the intestinal lumen.

## 4. Conclusions

Co-ingestion of black carrot and strawberry did not negatively affect the stability or bioaccessibility of the cyanidin-based anthocyanins monitored in the present study. In fact, an increased intestinal stability and enhanced bioaccessibility was found for specific-cyanidins as a consequence of co-ingestion. Therefore, the presence of strawberry food matrix positively affects cyanidin-based anthocyanin bioaccessibility, although no reciprocal benefits were observed for pelargonidin-based derivatives under the presence of black carrot food matrix. Pelargonidin-3-glucoside was the only anthocyanin negatively affected by co-digestion in terms of bioaccessibility in the small intestine, but, at the same time, this means that a higher amount of this anthocyanin would reach the lower parts of digestive tract, where it can also exert its beneficial effects. Cyanidin-3-xylosyl-glucosyl-galactoside stood out as the bioactive with higher overall stability and bioaccessibility values, under both single digestion (86% and 62%, for intestinal stability and bioaccesibility, respectively) or co-digestion conditions (99% and 105%, for intestinal stability and bioaccesibility, respectively), although it is suggested that cyanidin-3-xylosyl-glucosyl-galactoside might be obtained by removal of the feruloyl and coumaroyl from cyanidin-3-xylosyl-feruloyl-glucosyl-galactoside and cyanidin-3-xylosyl-coumaroyl-glucosyl-galactoside, respectively. The transport of all the studied cyanidin and pelargonidin-derivatives was maintained after the administration of the blended plant-based material (1.9–9.1%, after 4 h).

Taking into account the increasing consumer demands for fruit and vegetable mixtures (i.e., in the form of juices or smoothies) as a source of health-related compounds, a deep understanding of food components interactions is still an issue for food processors. This work adds valuable knowledge to the field and highlights the need for further mechanistic research to optimize mixtures that maximize the nutritional value of the final product.

## Figures and Tables

**Table 1:** Identification and quantification of black carrot and strawberry anthocyanins.

Peak	Mass (*m/z*)	Compound Identity	Source	mg/g dw
1	743	Cyanidin-3-xylosyl-glucosyl-galactoside	Black carrot	0.48 ± 0.05
2	581	Cyanidin-3-xylosyl-galactoside	Black carrot	1.35 ± 0.08
3	949	Cyanidin-3-xylosyl-sinapoyl-glucosyl-galactoside	Black carrot	2.16 ± 0.01
4	919	Cyanidin-3-xylosyl-feruloyl-glucosyl-galactoside	Black carrot	10.99 ± 0.02
5	889	Cyanidin-3-xylosyl-coumaroyl-glucosyl-galactoside	Black carrot	2.02 ± 0.01
6	433	Pelargonidin-3-glucoside	Strawberry	7.14 ± 0.01
7	519	Pelargonidin-3-malonyl-glucoside	Strawberry	2.24 ± 0.01

Peak numbers correspond to Figure 1. “dw” refers to dry weight. Concentrations expressed as mean ± SD (*n* = 2).

**Table 2:** Effect of co-digestion on gastric and intestinal stability of black carrot and strawberry anthocyanins, incubated as liquid extracts. Concentration expressed as mg/100 mL of extract (mean ± SD) (*n* = 3). Values in brackets represents the stability (%).

	Cyanidin-3-xylosyl-glucosyl-galactoside	Cyanidin-3-xylosyl-galactoside	Cyanidin-3-xylosyl-feruloyl-glucosyl-galactoside	Cyanidin-3-xylosyl-coumaroyl-glucosyl-galactoside	Pelargonidin-3-glucoside	Pelargonidin-3-malonyl-glucoside
	*Undigested*					
	0.42 ± 0.04	1.18 ± 0.07	9.60 ± 0.02	1.76 ± 0.01	12.31 ± 0.01	3.87 ± 0.02
	*Post-Gastric*					
Single digestion	0.25 ± 0.01 (59%) *a*	0.95 ± 0.00 (81%) *a*	8.23 ± 0.04 (86%) *a*	1.14 ± 0.01 (65%) *a*	11.13 ± 0.09 (90%) *a*	3.37 ± 0.01 (87%) *b*
Co-digestion	0.24 ± 0.02 (56%) *a*	0.98 ± 0.02 (83%) *a*	8.28 ± 0.03 (86%) *a*	1.16 ± 0.02 (66%) *a*	12.63 ± 0.46 (76%) *b*	3.57 ± 0.07 (92%) *a*
	*Post-Intestinal*					
Single digestion	0.36 ± 0.00 (86%) *b*	0.94 ± 0.01 (79%) *b*	5.54 ± 0.05 (58%) *b*	0.85 ± 0.07 (48%) *a*	6.01 ± 0.02 (49%) *a*	1.77 ± 0.01 (46%) *b*
Co-digestion	0.42 ± 0.00 (99%) *a*	1.09 ± 0.01 (92%) *a*	5.81 ± 0.03 (60%) *a*	0.75 ± 0.04 (42%) *a*	5.78 ± 0.13 (35%) *b*	1.91 ± 0.02 (49%) *a*

Different letters indicate significant differences in anthocyanin stability between single digestion and co-digestion, for each anthocyanin. To calculate pelargonidin-3-glucoside stability under co-digestion, the corresponding undigested value was considered as the sum of peak 3 + 6 (16.71 ± 0.06 mg/100 mL), as explained in Section 3.1.

**Table 3:** Effect of co-digestion on black carrot and strawberry anthocyanins bioaccesibility, incubated as matrix-containing powder. Concentration expressed as mg/100 g of sample (dry weight) (mean ± SD) (*n* = 3). Values in brackets represents bioaccessibility (%).

	Cyanidin-3-xylosyl-glucosyl-galactoside	Cyanidin-3-xylosyl-galactoside	Cyanidin-3-xylosyl-feruloyl-glucosyl-galactoside	Cyanidin-3-xylosyl-coumaroyl-glucosyl-galactoside	Pelargonidin-3-glucoside	Pelargonidin-3-malonyl-glucoside
	*Undigested*					
	48.2 ± 5.0	135.3 ± 7.6	1099.5 ± 2.0	201.7 ± 0.9	714.0 ± 0.5	224.2 ± 1.0
	*Post-digestion*					
Single digestion	29.7 ± 3.7 (62%) *b*	44.1 ± 4.4 (33%) *b*	378.4 ± 26.1 (34%) *b*	55.8 ± 5.2 (28%) *b*	256.6 ± 8.3 (36%) *a*	76.2 ± 1.8 (34%) *a*
Co-digestion	50.5 ± 9.2 (105%) *a*	113.8 ± 0.2 (84%) *a*	528.3 ± 15.0 (48%) *a*	105.2 ± 0.9 (52%) *a*	272.5 ± 19.7 (28%) *b*	81.3 ± 6.6 (36%) *a*

Different letters indicate significant differences in anthocyanin bioaccessibility between single digestion and co-digestion, for each anthocyanin. To calculate pelargonidin-3-glucoside bioaccessibility under co-digestion, the corresponding undigested value was considered as the sum of peak 3 + 6 (968.7 ± 3.7 mg/100 g), as explained in Section 3.1.

**Table 4:** Basal side recoveries of black carrot (BC) and strawberry (ST) anthocyanins and their mixture (Mix BC-ST) after 2 and 4 h of administration. Concentration expressed as µM (mean ± SD) (*n* = 3). Values in brackets represents the basal recovery (%).

Anthocyanin	Source	0 h	2 h	4 h
Cyanidin-3-xylosyl-glucosyl-galactoside	BC	3.1 ± 0.0	nd	0.28 ± 0.02 (9.0%) *a*
	Mix BC-ST	3.0 ± 0.0	nd	0.27 ± 0.01 (9.1%) *a*
Cyanidin-3-xylosyl-galactoside	BC	11.9 ± 0.1	0.28 ± 0.03 (2.3%) *a*	0.52 ± 0.07 (4.4%) *a*
	Mix BC-ST	11.7 ± 0.0	0.29 ± 0.01 (2.5%) *a*	0.48 ± 0.03 (4.1%) *a*
Cyanidin-3-xylosyl-feruloyl-glucosyl-galactoside	BC	91.6 ± 1.2	0.56 ± 0.12 (0.6%) *a*	1.93 ± 0.44 (2.1%) *a*
	Mix BC-ST	88.9 ± 0.3	0.52 ± 0.05 (0.6%) *a*	1.67 ± 0.18 (1.9%) *a*
Cyanidin-3-xylosyl-coumaroyl-glucosyl-galactoside	BC	13.5 ± 0.2	0.27 ± 0.02 (2.0%) *a*	0.47 ± 0.07 (3.5%) *a*
	Mix BC-ST	13.1 ± 0.0	0.28 ± 0.02 (2.2%) *a*	0.43 ± 0.04 (3.3%) *a*
Pelargonidin-3-glucoside	ST	81.0 ± 1.0	0.69 ± 0.17 (0.9%) *a*	1.94 ± 0.53 (2.4%) *a*
	Mix BC-ST	138.1 ± 2.1	0.53 ± 0.12 (0.4%) *a*	2.56 ± 0.31 (1.9%) *a*
Pelargonidin-3-malonyl-glucoside	ST	21.8 ± 0.1	nd	0.60 ± 0.07 (2.7%) *a*
	Mix BC-ST	24.2 ± 0.1	nd	0.46 ± 0.07 (1.9%) *a*

Different letters indicate significant differences in anthocyanin recovery between single and co-administration of black carrot and strawberry extract, for each anthocyanin. “nd” refers to none detected.

## References

[1] Kong J.-M., Chia L.-S., Goh N.-K., Chia T.-F., Brouillard R. (2003). Analysis and biological activities of anthocyanins. Phytochemistry.

[2] Castañeda-Ovando A., Pacheco-Hernández M.d.L., Páez-Hernández M.E., Rodríguez J.A., Galán-Vidal C.A. (2009). Chemical studies of anthocyanins: A review. Food Chem..

[3] Andersen Ø.M., Jordheim M., Andersen Ø.M., Markham K.R. (2006). The anthocyanins. Flavonoids: Chemistry, Biochemistry and Applications.

[4] de Mejia E.G., Zhang Q., Penta K., Eroglu A., Lila M.A. (2020). The Colors of Health: Chemistry, 560 Bioactivity, and Market Demand for Colorful Foods and Natural Food Sources of Colorants. Annu. Rev. Food Sci. Technol..

[5] Porrini M., Riso P. (2008). Factors influencing the bioavailability of antioxidants in foods: A critical appraisal. Nutr. Metab. Cardiovasc. Dis..

[6] Parada J., Aguilera J.M. (2007). Food microstructure affects the bioavailability of several nutrients. J. Food Sci..

[7] Bento-Silva A., Koistinen V.M., Mena P., Bronze M.R., Hanhineva K., Sahlstrøm S., Kitrytė V., Moco S., Aura A.-M. (2020). Factors affecting intake, metabolism and health benefits of phenolic acids: Do we understand individual variability?. Eur. J. Nutr..

[8] Bohn T., Carriere F., Day L., Deglaire A., Egger L., Freitas D., Golding M., Le Feunteun S., Macierzanka A., Menard O. (2018). Correlation between in vitro and in vivo data on food digestion. What can we predict with static in vitro digestion models?. Crit. Rev. Food Sci. Nutr..

[9] Zoubiri L., Bakir S., Barkat M., Carrillo C., Capanoglu E. (2019). Changes in the phenolic profile, antioxidant capacity and in vitro bioaccessibility of two Algerian grape varieties, Cardinal and Dabouki (Sabel), during the production of traditional sun-dried raisins and homemade jam. J. Berry Res..

[10] Kamiloglu S. (2019). Effect of different freezing methods on the bioaccessibility of strawberry polyphenols. Int. J. Food Sci. Technol..

[11] Kamiloglu S., Ozkan G., Isik H., Horoz O., Van Camp J., Capanoglu E. (2017). Black carrot pomace as a source of polyphenols for enhancing the nutritional value of cake: An in vitro digestion study with a standardized static model. LWT.

[12] Tomas M., Beekwilder J., Hall R.D., Sagdic O., Boyacioglu D., Capanoglu E. (2017). Industrial processing versus home processing of tomato sauce: Effects on phenolics, flavonoids and in vitro bioaccessibility of antioxidants. Food Chem..

[13] Carrillo C., Buvé C., Panozzo A., Grauwet T., Hendrickx M. (2017). Role of structural barriers in the in vitro bioaccessibility of anthocyanins in comparison with carotenoids. Food Chem..

[14] Gui-Fang D., Lei N., Wen A., Yuan-Huan W., Rui-Fang S., Xiu-Juan D., Hua-Bin L. (2019). Effects of simulated gastrointestinal digestion on antioxidant activities of individual and mixed fruits. N. Am. J. Med. Sci..

[15] Phan M.A.T., Bucknall M.P., Arcot J. (2019). Co-ingestion of red cabbage with cherry tomato enhances digestive bioaccessibility of anthocyanins but decreases carotenoid bioaccessibility after simulated in vitro gastro-intestinal digestion. Food Chem..

[16] Giusti M.M., Wrolstad R.E. (2003). Acylated anthocyanins from edible sources and their applications in food systems. Biochem. Eng. J..

[17] Stintzing F.C., Stintzing A.S., Carle R., Frei B., Wrolstad R.E. (2002). Color and antioxidant properties of cyanidin-based anthocyanin pigments. J. Agric. Food Chem..

[18] Kamiloglu S., Pasli A., Ozcelik B., Van Camp J., Capanoglu E. (2015). Colour retention, anthocyanin stability and antioxidant capacity in black carrot (*Daucus carota*) jams and marmalades: Effect of processing, storage conditions and in vitro gastrointestinal digestion. J. Funct. Foods.

[19] Kamiloglu S., Grootaert C., Capanoglu E., Ozkan C., Smagghe G., Raes K., Van Camp J. (2017). Anti-inflammatory potential of black carrot (*Daucus carota* L.) polyphenols in a co-culture model of intestinal Caco-2 and endothelial EA.hy926 cells. Mol. Nutr. Food Res..

[20] Minekus M., Alminger M., Alvito P., Ballance S., Bohn T., Bourlieu C., Carrière F., Boutrou R., Corredig M., Dupont D. (2014). A standardised static in vitro digestion method suitable for food—An international consensus. Food Funct..

[21] Buvé C., Kebede B.T., De Batselier C., Carrillo C., Pham H.T.T., Hendrickx M., Grauwet T., Van Loey A. (2018). Kinetics of colour changes in pasteurised strawberry juice during storage. J. Food Eng..

[22] Montilla E.C., Arzaba M.R., Hillebrand S., Winterhalter P. (2011). Anthocyanin composition of black carrot (*Daucus carota* ssp. *sativus* var. *atrorubens* Alef.) cultivars Antonina, Beta Sweet, Deep Purple, and Purple Haze. J. Agric. Food Chem..

[23] Algarra M., Fernandes A., Mateus N., de Freitas V., Esteves da Silva J.C.G., Casado J. (2014). Anthocyanin profile and antioxidant capacity of black carrots (*Daucus carota* L. ssp. *sativus* var. *atrorubens* Alef.) from Cuevas Bajas, Spain. J. Food Compos. Anal..

[24] Fang J. (2015). Classification of fruits based on anthocyanin types and relevance to their health effects. Nutrition.

[25] Dzhanfezova T., Barba-Espín G., Müller R., Joernsgaard B., Hegelund J.N., Madsen B., Larsen D.H., Martínez Vega M., Toldam-Andersen T.B. (2020). Anthocyanin profile, antioxidant activity and total phenolic content of a strawberry (*Fragaria × ananassa* Duch) genetic resource collection. Food Biosci..

[26] Marhuenda J., Alemán M.D., Gironés-Vilaplana A., Pérez A., Caravaca G., Figueroa F., Mulero J., Zafrilla P. (2016). Phenolic composition, antioxidant activity, and in vitro availability of four different berries. J. Chem..

[27] Kosińska-Cagnazzo A., Diering S., Prim D., Andlauer W. (2015). Identification of bioaccessible and uptaken phenolic compounds from strawberry fruits in in vitro digestion/Caco-2 absorption model. Food Chem..

[28] Josuttis M., Carlen C., Crespo P., Nestby R., Toldam-Andersen T.B., Dietrich H., Krüger E. (2012). A comparison of bioactive compounds of strawberry fruit from Europe affected by genotype and latitude. J. Berry Res..

[29] Stintzing F.C., Trichterborn J., Carle R. (2006). Characterisation of anthocyanin–betalain mixtures for food colouring by chromatic and HPLC-DAD-MS analyses. Food Chem..

[30] Kammerer D., Schillmöller S., Maier O., Schieber A., Carle R. (2007). Colour stability of canned strawberries using black carrot and elderberry juice concentrates as natural colourants. Eur. Food Res. Technol..

[31] McDougall G.J., Fyffe S., Dobson P., Stewart D. (2005). Anthocyanins from red wine-their stability under simulated gastrointestinal digestion. Phytochemistry.

[32] Pérez-Vicente A., Gil-Izquierdo A., García-Viguera C. (2002). In vitro gastrointestinal digestion study of pomegranate juice phenolic compounds, anthocyanins, and vitamin C. J. Agric. Food Chem..

[33] McDougall G.J., Fyffe S., Dobson P., Stewart D. (2007). Anthocyanins from red cabbage-stability to simulated gastrointestinal digestion. Phytochemistry.

[34] Yang Z., Tang C., Zhang J., Zhou Q., Zhang Z. (2019). Stability and antioxidant activity of anthocyanins from purple sweet potato (*Ipomoea batatas* L. cultivar Eshu No. 8) subjected to simulated in vitro gastrointestinal digestion. Int. J. Food Sci. Technol..

[35] Kammerer D., Carle R., Schieber A. (2004). Quantification of anthocyanins in black carrot extracts (*Daucus carota* ssp. *sativus* var. *atrorubens* Alef.) and evaluation of their color properties. Eur. Food Res. Technol..

[36] Bermúdez-Soto M.-J., Tomás-Barberán F.-A., García-Conesa M.-T. (2007). Stability of polyphenols in chokeberry (*Aronia melanocarpa*) subjected to in vitro gastric and pancreatic digestion. Food Chem..

[37] Fernandes I., Faria A., Calhau C., de Freitas V., Mateus N. (2014). Bioavailability of anthocyanins and derivatives. J. Funct. Foods.

[38] Fleschhut J., Kratzer F., Rechkemmer G., Kulling S.E. (2006). Stability and biotransformation of various dietary anthocyanins in vitro. Eur. J. Nutr..

[39] McDougall G.J., Dobson P., Smith P., Blake A., Stewart D. (2005). Assessing potential bioavailability of raspberry anthocyanins using an in vitro digestion system. J. Agric. Food Chem..

[40] Charron C.S., Kurilich A.C., Clevidence B.A., Simon P.W., Harrison D.J., Britz S.J., Baer D.J., Novotny J.A. (2009). Bioavailability of anthocyanins from purple carrot juice: Effects of acylation and plant matrix. J. Agric. Food Chem..

[41] Singh A., Kitts D.D. (2019). In vitro Bioaccessibility of Tart Cherry Anthocyanins in a Health Supplement Mix Containing Mineral Clay. J. Food Sci..

[42] Padayachee A., Netzel G., Netzel M., Day L., Mikkelsen D., Gidley M.J. (2013). Lack of release of bound anthocyanins and phenolic acids from carrot plant cell walls and model composites during simulated gastric and small intestinal digestion. Food Funct..

[43] Jakobek L., Matić P. (2019). Non-covalent dietary fiber—Polyphenol interactions and their influence on polyphenol bioaccessibility. Trends Food Sci. Technol..

[44] Bobrich A., Fanning K.J., Rychlik M., Russell D., Topp B., Netzel M. (2014). Phytochemicals in Japanese plums: Impact of maturity and bioaccessibility. Food Res. Int..

[45] Podsȩdek A., Redzynia M., Klewicka E., Koziołkiewicz M. (2014). Matrix effects on the stability and antioxidant activity of red cabbage anthocyanins under simulated gastrointestinal digestion. Biomed. Res. Int..

[46] Toydemir G., Boyacioglu D., Capanoglu E., Van Der Meer I.M., Tomassen M.M.M., Hall R.D., Mes J.J., Beekwilder J. (2013). Investigating the transport dynamics of anthocyanins from unprocessed fruit and processed fruit juice from sour cherry (*Prunus cerasus* L.) across intestinal epithelial cells. J. Agric. Food Chem..

[47] Kurilich A.C., Clevidence B.A., Britz S.J., Simon P.W., Novotny J.A. (2005). Plasma and urine responses are lower for acylated vs nonacylated anthocyanins from raw and cooked purple carrots. J. Agric. Food Chem..

[48] Dudonné S., Dal-Pan A., Dubé P., Varin T.V., Calon F., Desjardins Y. (2016). Potentiation of the bioavailability of blueberry phenolic compounds by co-ingested grape phenolic compounds in mice, revealed by targeted metabolomic profiling in plasma and feces. Food Funct..

[49] Kamiloglu S., Capanoglu E., Grootaert C., Van Camp J. (2015). Anthocyanin absorption and metabolism by human intestinal Caco-2 cells-a review. Int. J. Mol. Sci..

[50] Alzaid F., Cheung H.-M., Preedy V.R., Sharp P.A. (2013). Regulation of glucose transporter expression in human intestinal Caco-2 cells following exposure to an anthocyanin-rich berry extract. PLoS ONE.

[51] Fang J. (2014). Some anthocyanins could be efficiently absorbed across the gastrointestinal mucosa: Extensive presystemic metabolism reduces apparent bioavailability. J. Agric. Food Chem..

